# Dual-phase SilMA hydrogel: a dynamic scaffold for sequential drug release and enhanced spinal cord repair via neural differentiation and immunomodulation

**DOI:** 10.3389/fbioe.2024.1501488

**Published:** 2024-11-21

**Authors:** Ruizhi Zhang, Mingzhe Zhang, Lu Chen, Linlin Jiang, Chenbo Zou, Na Li, Hengxing Zhou, Shiqing Feng

**Affiliations:** ^1^ Department of Orthopaedics, Qilu Hospital, Shandong University Centre for Orthopaedics, Advanced Medical Research Institute, Cheeloo College of Medicine, Shandong University, Jinan, Shandong, China; ^2^ The 960th Hospital of the Joint Logistics Support Force of the Chinese People's Liberation Army, Jinan, China; ^3^ Center for Reproductive Medicine, Shandong University, Jinan, Shandong, China; ^4^ Department of Orthopaedics, The Second Hospital, Cheeloo College of Medicine, Shandong University, Jinan, Shandong, China

**Keywords:** spinal cord injury, SilMA hydrogel, neurotrophin-3, angiotensin-(1–7), immunomodulation, neural differentiation

## Abstract

**Introduction:**

Spinal cord injury (SCI) is a severe central nervous system disorder that results in significant sensory, motor, and autonomic dysfunctions. Current surgical techniques and high-dose hormone therapies have not achieved satisfactory clinical outcomes, highlighting the need for innovative therapeutic strategies.

**Methods:**

In this study, we developed a Dual-Phase Silk Fibroin Methacryloyl (SilMA) hydrogel scaffold (DPSH) that incorporates PLGA microspheres encapsulating neurotrophin-3 (NT-3) and angiotensin (1-7) (Ang-(1–7)). The DPSH is designed for temporally controlled release of therapeutic agents to reduce inflammation during the acute phase of SCI and to promote neuronal differentiation and axonal regeneration in later stages.

**Results:**

Comprehensive characterization of the DPSH revealed a highly porous architecture, suitable mechanical properties for spinal cord tissue, and stability unaffected by the incorporation of microspheres and drugs. In vitro studies demonstrated that Ang-(1–7) significantly induced M2 microglia polarization by 1.8-fold (p < 0.0001), effectively reducing inflammation. Additionally, NT-3 enhanced neural stem cell differentiation into neurons by 3.6-fold (p < 0.0001). In vivo experiments showed that the DPSH group exhibited significantly higher Basso Mouse Scale (BMS) scores (p < 0.0001), enhanced motor function, reduced astrocyte scarring by 54% (p < 0.05), and improved neuronal survival and regeneration.

**Discussion:**

These findings underscore the therapeutic potential of the DPSH scaffold for SCI repair, presenting a novel strategy to enhance neural recovery through a combination of immunomodulation and neuroprotection.

## 1 Introduction

Spinal cord injury (SCI) is a severe central nervous system disorder with a poor prognosis, often resulting from traumatic incidents such as traffic accidents or falls from heights. It can also be caused by inflammation or tumors. SCI can lead to significant sensory, motor, or autonomic dysfunctions, drastically reducing patients’ quality of life and imposing a heavy economic burden on families and society. In mammals, SCI triggers numerous pathological changes, including blood-brain barrier dysfunction, thrombosis, and neuronal death (Silva et al., 2021; Silva et al., 2014). Currently, clinical treatments for SCI include surgical and pharmacological approaches, but their effectiveness is limited. Surgical treatment cannot fully restore the anatomical structure of the damaged spinal cord and only addresses part of the primary injury, while oral or intravenous medications face challenges crossing the blood-spinal cord barrier to produce significant effects (Sanchez-Dengra et al., 2023; Shultz and Zhong, 2021).

Cell transplantation and *in situ* drug delivery are more effective strategies, providing cellular and nutritional support to the injured area (Lai et al., 2019; Ma et al., 2018). However, due to the lack of connectivity in the damaged region, transplanted cells or drugs struggle to establish cycles, preventing them from exerting their full potential (Sensharma et al., 2017).

SCI progresses through primary and secondary phases. The primary injury results from the initial trauma, causing mechanical disruption of the spinal cord’s anatomical continuity, blood-spinal barrier damage, local edema, hemorrhage, ischemia, inflammatory cell infiltration, and axonal demyelination. The secondary injury, triggered by the primary injury, involves continued edema, ischemia, inflammatory cell infiltration, further cell death, and the release of toxic substances (Jin et al., 2021; Zhou et al., 2023). These processes promote the formation of cystic cavities and glial scars, which hinder axonal regeneration and functional recovery (McDonald and Sadowsky, 2002; Ahuja et al., 2017; O'Shea et al., 2017; Sofroniew, 2018).

Microglia, the central nervous system’s macrophages, activate in response to SCI, re-leasing chemokines that attract immune cells to the injury site and exacerbate local dam-age. Activated microglia can polarize into M1 (pro-inflammatory) and M2 (anti-inflammatory) phenotypes. M1 microglia secrete TNF-α and IL-6, inducing inflammatory cascades, while M2 microglia secrete IL-10 and IL-4, alleviating inflammation and promoting repair (Orihuela et al., 2016). Currently, it is believed that cell death and scar formation due to an excessive local inflammatory response after spinal cord injury are significant barriers to nerve repair. Therefore, reducing M1 polarization of microglial cells and promoting M2 polarization after the injury to inhibit the local inflammatory response is a crucial research direction in spinal cord injury repair (Okada et al., 2022).

Studies show that anti-inflammatory factors such as IL-4, IL-10, and Ang-(1–7) can reduce inflammatory cell activation and acute inflammatory responses early in SCI, creating a favorable environment for repair (Saraiva and O'Garra, 2010; He et al., 2020; Pan et al., 2021; Gu et al., 2023). Neurotrophic factors like paclitaxel, NT-3, and NT-4 promote neuronal differentiation and axonal regeneration (NGF, 2014; Bao et al., 2022; Yan et al., 2023; Fukuyama et al., 2024). However, endogenous repair mechanisms often fall short due to insufficient recruitment of endogenous neural stem cells and excessive differentiation into astrocytes, forming glial scars that impede repair (Murk et al., 2013; Blanco-Suarez et al., 2017; Zhou et al., 2019; Endo et al., 2022; Hellenbrand et al., 2021).

Hydrogels are a polymer material with a three-dimensional network structure, using water as the dispersion medium, which is both biocompatible and degradable (Yuan et al., 2021). Hydrogel itself can mimic the natural extracellular matrix to fill the damaged area and improve the microenvironment of the damaged area. It can also act as a carrier for seed cells and active factors, helping seed cells to colonize and proliferate, promoting the reconnection of damaged spinal cord tissues, helping to bridge the gap at the injury site and re-establishing nerve conduction, and achieving sustained drug release. In current tissue engineering research for spinal cord injury treatment, hydrogel primarily serves the following roles: First, it provides three-dimensional spatial support for neuronal regeneration and axonal extension, facilitating cell growth, proliferation, and migration, and promoting neural reconstruction (Chedly et al., 2017; Silva et al., 2023); Second, hydrogel acts as a carrier, delivering stem cells to the injury site, thereby reducing cell loss (Peng et al., 2023). Third, hydrogel slowly releases bioactive factors or chemical drugs, ensuring continuous and stable release for improved therapeutic effects (Luo et al., 2021). Additionally, proteins or extracellular matrix components such as hyaluronic acid and filipin proteins, known for their biocompatibility and tissue affinity, and suitability for chemical and structural modifications (Gao et al., 2022), have also been used to synthesize hydrogel scaffolds. Currently, synthetic hydrogel raw materials commonly used in tissue engineering include polyacrylic acid and its derivatives, polyvinyl alcohol, and polyester (Anderson et al., 2018; Ranganathan et al., 2018; Han et al., 2022; Cai et al., 2023; Xiao et al., 2023).

Silk fibroin (SF) derived from processed silkworm silk is a promising biomaterial with excellent degradability, biocompatibility, and high tensile strength (Wen et al., 2016; Hoque et al., 2019). However, its water-insolubility and limited processability can restrict its applications. Methacrylated silk fibroin (SilMA), a modified form of SF, addresses these limitations by offering improved water solubility and the ability to be crosslinked into a gel under UV light. UV crosslinking allows for precise control over the gelation process, enabling tunable mechanical properties and rapid fabrication, making it particularly advantageous for biomedical applications. This approach also facilitates the incorporation of bioactive molecules and cells during gel formation, preserving their functionality. SilMA hydrogels combine the structural benefits of silk fibroin with the functional properties of hydrogel materials, such as controlled degradation and mechanical adaptability, making them a highly versatile and promising option for tissue engineering and regenerative medicine.

This study constructs a Dual-Phase SilMA hydrogel scaffold (DPSH), incorporating PLGA microspheres encapsulating neurotrophin-3 (NT-3) and angiotensin (Silva et al., 2021; Silva et al., 2014; Sanchez-Dengra et al., 2023; Shultz and Zhong, 2021; Lai et al., 2019; Ma et al., 2018; Sensharma et al., 2017) (Ang-(1–7), and neural stem cells. The hydrogel’s degradation, in conjunction with PLGA microspheres, enables sequential drug release. Ang-(1–7) is released early to reduce inflammation and protect neurons, followed by the release of neurotrophic factors to promote neuronal differentiation and axonal regeneration. This precise regulation aims to neural function recovery post-SCI, offering a novel therapeutic approach.

## 2 Materials and methods

### 2.1 Experimental animals

Experimental animals were purchased from Beijing Viton Lihua Company, 6–8 weeks old C57BL/6J female mice, weighing 18–25 g. All animal procedures were approved by the Ethics Committee of Shandong University (Approval No. ecsbmssdu24019). During the experiment, the mice were housed in the SPF-grade animal room of Shandong University Experimental Animal Center with sufficient feed and water. The mice could freely eat, the temperature was set at 20°C–25°C, and the relative humidity was set at 40%–60%. The feeding and experimental handling process of the mice complied with the “Regulations on the Administration of Laboratory Animals” by the National Science and Technology Com-mission and fully followed the regulations of the Ethics Committee of Shandong University. All operators were trained according to relevant guidelines and regulations.

### 2.2 Experimental materials

The batch names, brands, and catalog numbers of all materials and reagents used in this study are detailed in Table 1.

**TABLE 1:** Materials used in the experiment.

Materials	Catalog number	Company name
DMEM	Cat#10566016	Gibco
B-27	Cat#17504044	Gibco
Penicillin-streptomycin	Cat#15140163	Gibco
Stem Pro Accutase	Cat#1110501	Gibco
papain	Cat#G8430	Solarbio
DNAse	Cat#D8071	Solarbio
PBS	Cat#P1022	Solarbio
FBS	Cat#SA101.02	CellMax
DAPI	Cat#C0065	Solarbio
Triton X-100	Cat#T8200	Solarbio
TWEEN-20	Cat#T8220	Solarbio
BSA	Cat#ST023	Beyotime
CCK-8	Cat#C0042	Beyotime
Calcein/Pl	Cat#C2015M	Beyotime
Anti-beta lIl Tubulin antibody	Cat#ab215037	Abcam
GFAP (GAS) Mouse mAb	Cat#3670	CST
SilMA	Cat# EFL-SilMA-001	Engineering For Life
Ang- (1–7)	Cat#12403	Sigma
LPS	Cat#L3024	Sigma
NT-3	Cat#N1905	Sigma

### 2.3 Preparation of SilMA hydrogel and PLGA microspheres

All SilMA hydrogels used in this study were purchased from Engineering For Life and were prepared for both *in vitro* and *in vivo* experiments through dissolution, filtration for sterilization, and UV cross-linking.

#### 2.3.1 Preparation of SilMA hydrogel

A 0.25% (w/v) initiator solution was prepared by dissolving 0.05 g LAP in 20 mL PBS, heating in a 40°C–50°C water bath for 15 min, and shaking occasionally. An 8% SilMA hydrogel solution was created by weighing the required amount of SilMA, placing it in a centrifuge tube, and adding the initiator solution. The solution was dissolved at room temperature for 30 min, with occasional stirring. The SilMA solution was sterilized using a 0.22 μm sterile needle filter in a clean bench. The SilMA solution was injected into well plates (50 μL/well for 96-well plates, 100 μL/well for 48-well plates, and 300 μL/well for 24-well plates) and was irradiated with a 405 nm light source for 30 s to gel. Culture medium was added to cover the gel, incubated at 37°C for 5 min, then washed and the medium was removed. A cell suspension was added for culturing. For *in vivo* hydrogel scaffolds, the filtered solution was cross-linked with light and trimmed to the appropriate size. To ensure that all hydrogels experienced the same amount of UV energy during crosslinking, a standardized UV exposure protocol was used. Specifically, a uniform UV intensity was applied using a calibrated light source positioned at a fixed distance from the samples, ensuring consistent energy delivery. Additionally, the exposure time was precisely controlled for all samples to maintain consistent crosslinking. This method minimized variability in gelation and mechanical properties, ensuring reproducibility across experiments.

#### 2.3.2 Preparation of PLGA microspheres

5 mg of NT-3 was dissolved in 1 mL deionized water (aqueous phase) and 100 mg of polylactic acid was dissolved in 4 mL dichloromethane and 1 mL acetone (organic phase). The aqueous and organic phases were mixed, then ultrasonicated in an ice bath at 100 W for 1 min to form a uniform dispersion. The mixture was slowly injected into 25 mL of 1% polyvinyl alcohol solution, and sonicated in an ice bath at 200 W for 3 min to form an emulsion. The emulsion was stirred at low speed (400 rpm) for 3–5 h to allow complete dispersion of dichloromethane and acetone. It was frozen and centrifuged at high speed (12000 rpm, 20 min) to separate the PLGA microspheres, which were washed three times with deionized water. The microspheres were freeze-dried, sterilized at 60°C, and stored at 4°C.

### 2.4 Preparation of DPSH scaffold

Using the same method as for SilMA hydrogel, an 8% hydrogel solution was prepared, dissolved thoroughly, and filtered for sterilization. Then, 10 μg/ml PLGA microspheres containing NT-3 and 5 μg/ml Ang-(1–7) were added to the neural stem cell pellet. The mixture was mixed well and injected into well plates, irradiated with a 405 nm light source for 30 s to gel. The same steps were followed for further processing.

### 2.5 Characterization of SilMA hydrogel and PLGA microspheres

#### 2.5.1 SilMA hydrogel characterization

The mechanical properties of the hydrogel were measured at 25°C using a HAAKE MARS rheometer, calculating the elastic modulus (G′) and viscous modulus (G″). Mechanical properties were analyzed with a GT TCS-2000 single-column tester. Samples (n = 3) were formed into cylinders of 10 mm diameter and 3 mm height and tested at a 1 mm/min compression speed to determine the Young’s modulus from the strain-stress curve slope. For the rheological characterization of the SilMA hydrogel, dynamic rheology experiments were conducted using a HAAKE MARS III photorheometer equipped with a parallel-plate geometry (P20 TiL, 20-mm diameter). Time-sweep oscillatory tests were performed under a 10% strain (in CD mode) with a frequency of 1 Hz and a 0.5 mm gap over 180 s.

#### 2.5.2 PLGA microspheres characterization

The size and morphology of PLGA microspheres were observed under a scanning electron microscope (SEM, Philips XL-30) at a 10 kV acceleration voltage.

#### 2.5.3 *In vitro* drug release

20 mg of each drug-loaded material was weighed, dissolved in 20 mL PBS (Wu et al., 2022), and incubated at 37°C on a constant temperature shaker. At specified intervals (1, 3, 7, 14, 21, 28, 35, 42, 49, and 56 days), the samples were centrifuged at 2000 rpm for 10 min, 1 mL of supernatant was removed, replaced with fresh PBS, and incubation continued. All supernatants were collected and drug content was measured using ELISA kits, plotting the drug release curve over time. This process has the potential to alter the drug concentration and the driving force for release. To ensure accuracy, we factored in both the amount of drug present in the removed PBS and the concentration remaining in the hydrogel. By doing so, we adjusted for any dilution effect caused by the PBS replenishment. This approach allowed us to accurately track the cumulative drug release over time, even as PBS was refreshed. The resulting release profile reflects the actual total drug release, maintaining the integrity of the study despite the replenishment process.

### 2.6 Cell culture

The water bath was preheated to 37°C. Frozen cells were retrieved from −80°C storage and thawed quickly in the water bath, shaking gently until ice crystals dissolved. The vial was disinfected with 75% alcohol, placed in a sterile workbench, and the cell suspension was transferred to a 10 mL centrifuge tube with 4 mL complete medium. The tube was centrifuged at 1000 rpm for 3 min, the supernatant was removed, and the cells were resuspended in 1 mL complete medium and plated in a 6 cm culture dish with 3 mL complete medium. When cells reached 70%–80% confluence, sterile PBS, trypsin, and complete medium were warmed in a 37°C water bath. The medium was removed, the cells were washed with 1 mL PBS twice, and 1 mL trypsin was added. The cells were incubated until they began to detach. Digestion was stopped with 2 mL complete medium, the cells were collected in a 10 mL centrifuge tube, and centrifuged at 1000 rpm for 3 min. The cells were resuspended in complete medium, replated, and incubated at 37°C, 5% CO2. When cells were in good condition, sterile PBS, trypsin, and complete medium were prepared as described. Cells were detached with trypsin, digestion was stopped, the cells were centrifuged, and resuspended in 900 µL fetal bovine serum and 100 µL DMSO. The suspension was transferred to cryovials, placed in a freezing container, and stored at −80°C.

### 2.7 Isolation and culture of mouse neural stem cells

Instruments and materials were sterilized, plates were prepared with poly-L-lysine, and all required solutions were readied in a sterile environment. Pregnant mice were euthanized, disinfected in 75% alcohol, and transferred to a sterile workbench. Embryos were extracted, and their heads were placed in pre-cooled high-glucose medium. The cerebral cortex was isolated, removing meninges and other tissues, and transferred to new dishes for mincing. The samples were centrifuged at 1200 rpm for 5 min, resuspended in papain and DNAse, digested at 37°C, and then centrifuged again. The solution was filtered through a 40μm mesh, and the cells were counted. Cells were plated in T75 flasks with proliferation medium, incubated at 37°C, 5% CO_2_, and the medium was changed as needed. Cells were passaged when neurospheres grew large and opaque. Finally, cells were plated in differentiation medium with hydrogel scaffolds in Transwell inserts.

### 2.8 CCK8 assay

Neural stem cells were plated in 96-well plates with varying concentrations of SilMA hydrogel solution. The cells were incubated for 3 days, after which the medium was replaced with medium containing 10% CCK8 reagent. The plates were incubated for 2 h, and absorbance was measured at 450 nm.

### 2.9 Microglia inflammation induction and staining

BV2 microglial cells were plated on poly-L-lysine-coated 24-well plates, and inflammation was induced using lipopolysaccharide (LPS) to activate the cells. Simultaneously, a Transwell system was used, with a drug-loaded hydrogel placed in the upper chamber and BV2 cells in the lower chamber, to assess the hydrogel’s impact on inflammation over 24 h. After 24 h of co-culture with the hydrogel, the BV2 cells were fixed, permeabilized, and blocked. The cells were incubated with primary antibodies (anti-iNOS for M1 macrophages, anti-ARG-1 for M2 macrophages) and then with fluorescently labeled secondary antibodies (488 anti-mouse, 555 anti-rabbit). Finally, the nuclei were stained with DAPI, and the cells were observed under a fluorescence microscope to evaluate the expression of inflammatory markers and the effect of the hydrogel on inflammation.

### 2.10 Live/dead cell staining

After culturing the cells with 1% SilMA hydrogel for 3 days, the cells were stained with Calcein AM/PI, incubated, and observed under a fluorescence microscope.

### 2.11 Neural stem cell differentiation and staining

To differentiate neural stem cells with hydrogel scaffolds, a Transwell system was used for a 7-day co-culture. The hydrogel scaffold was placed in the upper chamber, and neural stem cells were placed in the lower chamber, allowing for sustained release of therapeutic agents from the hydrogel while maintaining proximity to the cells. After 7 days of co-culture, the cells were fixed, permeabilized, and blocked. Next, the cells were incubated with primary antibodies (anti-Tuj-1 to identify neurons, anti-GFAP to identify astrocytes) to assess differentiation. Following primary antibody incubation, the cells were treated with appropriate secondary antibodies, their nuclei were stained with DAPI, and they were observed under a fluorescence microscope to evaluate the extent of neural differentiation and astrocyte formation.

### 2.12 *In vivo* degradation of hydrogel

Mice were acclimated, instruments were sterilized, and subcutaneous implantation of 100 μg hydrogel was performed. Post-surgery, antibiotics were administered, and hydrogel degradation was measured at set intervals.

### 2.13 Establishment of mouse SCI model

After a 1-week acclimatization period, mice were anesthetized with isoflurane (RWD, R510-22, Guangdong, China) to establish a spinal cord injury model. A longitudinal incision was made over the T10 vertebra, the muscles were gently separated, and the T9-T11 spinal segments were exposed. A complete laminectomy at T10 was performed, followed by a 2 mm complete transection of the spinal cord. Successful model establishment was confirmed, and the incision was sutured in layers.

### 2.14 Behavioral testing

BMS scores were evaluated using the BMS mouse scale guidelines at pre-SCI, day 1, day 7, and weekly up to 8 weeks post-SCI by three blinded observers. Prior to testing, mice were acclimatized to the testing environment. The open field was surrounded by a trans-parent glass plane, enabling observers to assess hind limb joint movement, weight sup-port, plantar stepping, and coordination. BMS scores ranged from 0 (no ankle movement) to 9 (full recovery) and were recorded to measure hind limb motor function.

### 2.15 Preparation of frozen sections

Mice were transcardially perfused with pre-cooled PBS until the effluent from the right atrium was clear and the liver appeared pale. Subsequently, pre-cooled 4% para-formaldehyde was perfused, and the spinal cord was harvested. Spinal cord tissues were fixed overnight at 4°C in 4% paraformaldehyde, dehydrated in sucrose solution, embedded in OCT, and sectioned at 10 μm thickness using a cryostat (Leica, CM3050S, Germany). Sections were cut at a thickness of 10 μm to balance tissue detail and structural integrity. The cryostat temperature was set at −20°C to prevent ice crystal formation and tissue damage.

### 2.16 Immunohistochemical staining

The frozen sections were rinsed with distilled water for 10 min to remove excess embedding medium. A histochemical pen was used to outline the areas for staining. The sections were placed in a humid chamber, and a blocking and permeabilization solution containing 5% BSA and 0.5% Triton X-100 was added. The sections were incubated at room temperature for 1 h to block non-specific antigen sites and permeabilize the cells. After blocking, primary antibodies, including anti-Tuj-1 antibody (1:200), anti-GFAP antibody (1:200), anti-iNOS antibody (1:200), and anti-Arg-1 antibody (1:200), were applied to the sections. The sections were covered with a coverslip and were incubated overnight at 4°C. The next day, the sections were washed three times with TBST solution for 5 min each. Fluorescent secondary antibodies, including 488 anti-mouse antibody (1:400) and 555 anti-rabbit antibody (1:400), were applied to the sections, followed by a 1-h incubation at room temperature in the dark. The sections were washed three times with TBST solution for 5 min each to remove residual secondary antibodies. An appropriate amount of DAPI-containing antifade mounting medium was added to the spinal cord sections. A coverslip was carefully placed on top, ensuring no air bubbles were trapped. The edges of the coverslip were sealed with nail polish. Immunofluorescence staining was observed under a fluorescence microscope, and images were captured for further analysis.

### 2.17 Hematoxylin and eosin staining

Frozen sections were rinsed with distilled water three times for 5 min each to remove excess embedding medium. The sections were placed in hematoxylin stain for 2 min, then were rinsed under running water until the tissue turned blue. The sections were dipped in 1% hydrochloric acid alcohol for approximately 3 s, then were rinsed under running water for 1 min. The sections were stained with eosin for 3 min, followed by a 1-min rinse under running water. Sequentially, the sections were placed in 80% ethanol, 95% ethanol, and absolute ethanol for 5 min each. The sections were transferred into xylene I and xylene II, each for 5 min. The sections were mounted with neutral resin and observed under a microscope.

### 2.18 Protein extraction

The lysis buffer, containing RIPA and PMSF in a 1000:1 ratio, was prepared and kept on ice. The culture medium was discarded, and the cells were rinsed with PBS. Lysis buffer was added to the cells, and they were incubated on ice for 10 min. The cells were scraped into 1.5 mL centrifuge tubes and were sonicated for 1 min. Fresh spinal cord tissue was collected from the injury site and adjacent segments (each 0.5 mm), placed in 1.5 mL centrifuge tubes on ice, lysis buffer was added, and the tissue was homogenized until no visible fragments remained. The lysates were incubated on ice for 10 min. The centrifuge was pre-cooled to 4°C, and the lysates were centrifuged at 12000 rpm for 30 min. The supernatant was transferred to a new tube, and the protein concentration was determined. Loading buffer was added, the solution was heated at 95°C for 10 min to denature the proteins, and the samples were stored at −20°C.

### 2.19 BCA protein assay

A series of BSA standards with an initial concentration of 5 mg/ml and final concentrations ranging from 0.078125 mg/ml were prepared. Reagent A and reagent B were mixed in a 50:1 ratio to prepare the BCA working reagent. 20 μL of each standard and protein sample were added to a 96-well plate, followed by 200 μL of BCA working reagent in each well. The mixture was incubated at 37°C for 30–40 min, avoiding light. Absorbance was measured at 562 nm using a microplate reader, and protein concentrations were calculated from the standard curve.

### 2.20 Western blot analysis

The gel apparatus and glass plates were assembled, filled with water, and checked for leaks. Once confirmed leak-free, the gel preparation was started. SDS-PAGE gels, appropriate for the target protein’s molecular weight, were prepared. The separating gel was poured between the glass plates and overlaid with water, then allowed to polymerize for 30–60 min. After removing the water, the stacking gel was added, and the comb was inserted. The gel was allowed to polymerize completely. Frozen protein samples were thawed on ice and loaded into the gel wells along with the protein ladder as a molecular weight marker. The gel was run at 80 V until the samples entered the separating gel, then the voltage was increased to 120 V until the dye front reached the bottom. The gel apparatus was disassembled, and the gel was trimmed. The PVDF membrane was activated in methanol. The transfer sandwich was assembled, and proteins were transferred at 100 V constant voltage. The membrane was blocked with 5% BSA in TBST for 1 h at room temperature to prevent non-specific binding. The membrane was incubated overnight at 4°C with primary antibodies (e.g., anti-β-actin, anti-iNOS, anti-Arg-1, anti-GFAP, anti-Tuj-1, all at 1:1000 dilution). The membrane was washed three times with TBST and incubated with HRP-conjugated secondary antibodies (goat anti-rabbit IgG or goat anti-mouse IgG, 1:10,000) for 1 h at room temperature. The ECL detection reagent was prepared according to the manufacturer’s instructions, applied to the membrane, and the protein bands were visualized using a chemiluminescence imaging system.

### 2.21 Statistical analysis

All data were analyzed with GraphPad Prism 8.0 (GraphPad Software, USA), and the values are presented as the mean ± standard error of the mean (SEM); each value represents the average of three independent experiments. Statistical significance was deter-mined by the independent sample *t*-test when two groups were being compared, by one-way analysis of variance (ANOVA) followed by Bonferroni *post hoc* analysis for multiple comparisons when three or more groups were being compared. Statistical significance is defined as *P* < 0.05, with * indicating *P* < 0.05, ** indicating *P* < 0.01, *** indicating *P* < 0.001, **** indicating *P* < 0.0001. Non-significant differences are marked as ns.

## 3 Results and discussion

### 3.1 Material characterization

**FIGURE 1:**
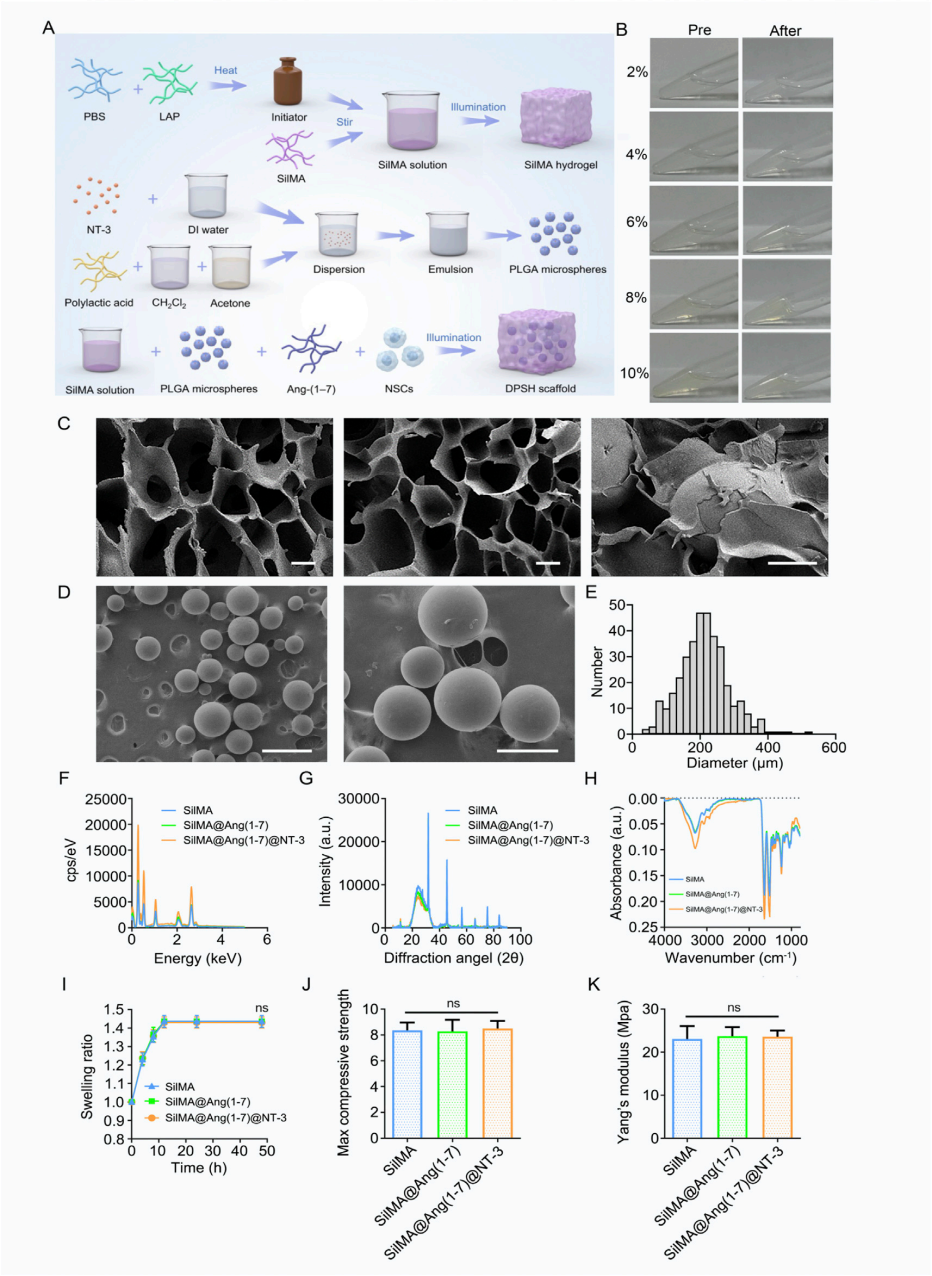
Preparation and Characterization of SilMA Hydrogel. **(A)** Schematic diagram of 4-Dimensional SilMA Hydrogel synthesis steps **(B)** Images of SilMA hydrogel before and after formation. **(C)** SEM images of SilMA hydrogel, scale bar = 20 μm. **(D)** SEM images of NT-3 encapsulated PLGA microspheres, scale bars = 500 μm (left) and 200 μm (right). **(E)** Particle size distribution of PLGA microspheres. **(F)** Energy profiles of SilMA hydrogels with different components. **(G)** X-ray diffraction patterns of SilMA hydrogels with different components. **(H)** Absorbance spectra of SilMA hydrogels with different components. **(I)** Swelling rates of SilMA hydrogels with different components. **(J)** Maximum compressive strength of SilMA hydrogels with different components. **(K)** Young’s modulus of SilMA hydrogels with different components.

The images showing appearance changes before and after hydrogel formation are presented in Figure 1B. To assess whether the structure of SilMA hydrogel is suitable for carrying drugs and cells for spinal cord injury repair, its microstructure was examined using scanning electron microscopy (SEM). The results showed that the SilMA hydrogel exhibits a porous network structure with high porosity (Figure 1C). This structure is capable of carrying drugs, cells, and microspheres, serving as a channel for material exchange within the body and providing the necessary space for injury repair.

Due to the excellent biocompatibility and biodegradability of poly (lactic-co-glycolic acid) (PLGA), PLGA microspheres were used in this study to encapsulate NT-3. This approach prevents the rapid release of NT-3 *in vivo*, ensuring its prolonged presence at the injury site, particularly maintaining high concentrations during the later stages of repair. SEM observations revealed that the microspheres were well-dispersed without aggregation (Figure 1D). Analysis of SEM images using ImageJ software determined that the size and particle size distribution were approximately 200 μm (Figure 1E). This indicates that the microspheres can be evenly distributed within the hydrogel and released gradually as the hydrogel degrades, thereby achieving sustained drug release. These results demonstrate that well-formed NT-3-loaded PLGA microspheres were successfully fabricated for this study.

We also tested the Energy, Intensity, Absorbance, and Swelling rate of the hydrogels, both with and without drug loading. The results showed no significant differences be-tween the drug-loaded hydrogels and the standard SilMA hydrogels (Figures 1F–I). X-ray diffraction (XRD) patterns further confirmed that the diffraction peak positions and intensities remained unchanged across the three hydrogel groups, indicating that the in-corporation of drugs and PLGA microspheres did not alter the protein structure of the SilMA hydrogels (Figure 1G). Additionally, the Young’s modulus and maximum com-pressive strength of the SilMA, SilMA/Ang-(1–7), and SilMA/Ang-(1–7)/NT-3 hydrogels showed no significant differences (Figures 1J,K). This suggests that the addition of drugs and PLGA microspheres does not adversely affect the mechanical properties of the hydrogels. In summary, the 8% concentration of SilMA hydrogel demonstrated the best structural integrity during handling and exhibited optimal mechanical properties suitable for further experimental applications. This concentration was robust enough to support subsequent cellular interactions and drug release studies while also ensuring adequate diffusion of therapeutic agents. Based on these visual observations and practical evaluations, the 8% concentration was chosen for all subsequent experiments, as it provided the right balance between handling ease and experimental consistency, making it ideal for both *in vitro* and *in vivo* settings.

### 3.2 Biocompatibility and cytotoxicity of SilMA hydrogel

**FIGURE 2:**
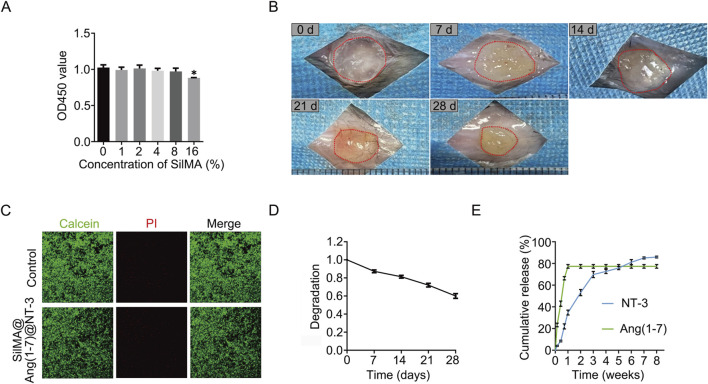
Degradation Rate, Sustained Release Capability, and Biocompatibility of SilMA Hydrogels. **(A)** CCK8 assay results of neural stem cells cultured with SilMA hydrogels at different concentrations. **(B)** Degradation images of SilMA hydrogels in mice over the first 4 weeks. **(C)** Live/dead staining results of neural stem cells co-cultured with SilMA hydrogels, scale bar = 100 μm. **(D)** Degradation curve of SilMA hydrogels in mice over the first 4 weeks. **(E)** Drug release curves of SilMA hydrogels loaded with Ang-(1–7) and NT-3 encapsulated in PLGA microspheres.

The biocompatibility of a material is a crucial factor influencing its potential applica-tions. The CCK8 assay is a commonly used method for analyzing cell proliferation. To determine whether SilMA affects the proliferation of neural stem cells, the cells were cultured in media containing different concentrations of SilMA under *in vitro* conditions. After 3 days of culture, the CCK8 reagent was added to assess cell proliferation. The results showed that only in the group with a SilMA concentration higher than 16% was the proliferation of neural stem cells inhibited. No statistically significant differences were observed between the lower concentration experimental groups and the control group (Figure 2A). This indicates that SilMA has good biocompatibility and confirms that using an 8% concentration in this study is safe.

Besides the effect on proliferation, it is crucial to assess whether the material causes cytotoxicity, leading to cell death, as this is a critical factor for the success of transplantation. Live/Dead staining is a method used to assess cell viability based on esterase activity and membrane integrity. Commercial kits facilitate this analysis using the dual fluorescence staining method of Calcein-AM (Calcein) and Propidium Iodide (PI). Calcein-AM stains live cells green, while PI stains dead cells red. Similar to the previous method, after 3 and 7 days of culture, no significant cell death was detected, indicating that SilMA has excellent biosafety and does not cause tissue cell death when implanted *in vivo* (Figure 2C).

### 3.3 Degradation of SilMA hydrogel

During the acute and subacute phases of spinal cord injury, the local environment undergoes significant changes, including severe bleeding, ischemia, and cell necrosis. Hydrogels implanted at the injury site can fill the damaged area and help stop bleeding, thereby reducing secondary damage. However, the degradation rate of the hydrogel is crucial for spinal cord repair. If it degrades too quickly, it will lose its function as a filler; if it degrades too slowly, it can interfere with normal tissue regeneration and impede repair. To assess the *in vivo* degradation of SilMA hydrogel, it was implanted subcutaneously in mice (n = 3 per group) and observed for 4 weeks. The results showed that the hydrogel gradually degraded over this period, reaching 62% of its original size by the fourth week (Figures 2B, D). This indicates that the SilMA hydrogel can provide long-term support during the spinal cord injury repair process. We initially chose subcutaneous injection for degradation studies due to its simplicity and standardization. However, recognizing that *in-situ* injection at the spinal cord injury site would more accurately represent real conditions, we will adapt this approach in future studies. Furthermore, While the 28-day results clearly showed that the hydrogel underwent significant degradation, leading to the effective release of Ang-(1–7) and achieving our early anti-inflammatory objectives, we recognize the importance of monitoring the hydrogel’s performance over a more extended period. Future studies will include longer-term degradation assessments, such as up to 8 weeks, to provide a more comprehensive understanding of the hydrogel’s stability and its sustained release profile, especially concerning the prolonged effects of NT-3 on neural regeneration. In general, PLGA degradation can take several weeks to months, providing a sustained release profile for encapsulated agents like NT-3. In our study, although we did not directly measure PLGA degradation rates, similar experiments (Jusu et al., 2020) suggests that PLGA with similar properties to what was used in our system undergoes gradual hydrolysis, which would release NT-3 over an extended period. This slow degradation is essential for ensuring the availability of NT-3 during the later stages of SCI repair, when neuronal differentiation and axonal regeneration are critical for functional recovery. By delivering NT-3 in a controlled and sustained manner, PLGA microspheres help maintain a neurotrophic environment, supporting long-term tissue regeneration.

### 3.4 Drug release of Ang-(1–7) and NT-3

The local accumulation of endogenous neurotrophic and anti-inflammatory factors is insufficient to mitigate the adverse effects of spinal cord injury, making drug delivery and release a critical aspect of the repair process. The release profiles of the two drugs incorporated into the SilMA hydrogel were assessed using ELISA kits. As shown in Figure 2E, the hydrogel released over 40% of Ang-(1–7) within the first 3 days and nearly all of it within 7 days. This indicates that the SilMA hydrogel has excellent substance ex-change capabilities, enabling a substantial early release of Ang-(1–7) to inhibit local inflammatory responses and improve the inflammatory microenvironment at the injury site. This helps reduce inflammation, protect nerve cells, and create a favorable environment for subsequent neural repair. Due to the encapsulation by PLGA, NT-3 was released slowly and continuously, with approximately 14% still being released between days 28 and 56. This sustained release of neurotrophic factors during the later stages of injury can promote the differentiation of neural stem cells into neurons and axonal regeneration, thereby facilitating the recovery of neural function. We chose PBS for the initial drug release study to maintain reproducibility and control over experimental variables. PBS provides a stable, physiologically relevant environment without introducing external biological factors such as enzymes or proteins, which could complicate the analysis. This allowed us to focus on understanding the fundamental release mechanisms of the drugs within the hydrogel. We acknowledge the limitations of using PBS as the release medium. While PBS is commonly used in in vitro studies for its simplicity and reproducibility, we recognize that it does not fully replicate the complex *in vivo* environment, particularly the oxidative stress and elevated levels of reactive oxygen species (ROS), such as H2O2, present in injury sites like the spinal cord. In future studies, we aim to address this by incorporating conditions that mimic the inflammatory and oxidative stress environments, such as using PBS supplemented with elevated H2O2 levels. Moving forward, we will integrate more physiologically relevant conditions into our experimental setup to enhance the applicability of our findings to clinical settings.

### 3.5 Ang-(1–7) promotes microglia polarization towards M2 phenotype in vitro

Local inflammatory responses after spinal cord injury are a major cause of cell death during the acute phase. The accumulation of inflammatory cytokines and infiltration of inflammatory cells not only lead to cell death but also contribute to the formation of spinal cord cysts from macrophage death during the subacute and chronic phases, severely hindering injury repair. Additionally, studies have shown that reducing the inflammatory response after spinal cord injury can decrease glial scar formation, promoting axonal re-generation and functional recovery of the spinal cord.

To investigate this, an *in vitro* model was established using lipopolysaccharide (LPS) to induce acute inflammation in mouse microglia (BV2 cells), simulating the acute in-flammatory process. LPS can polarize BV2 cells towards the M1 phenotype. The experimental group consisted of SilMA hydrogel loaded with Ang-(1–7), co-cultured with BV2 cells using Transwell inserts. Immunofluorescence staining and Western blot (WB) analysis were then performed to observe the effect of Ang-(1–7) on microglial polarization.

**FIGURE 3:**
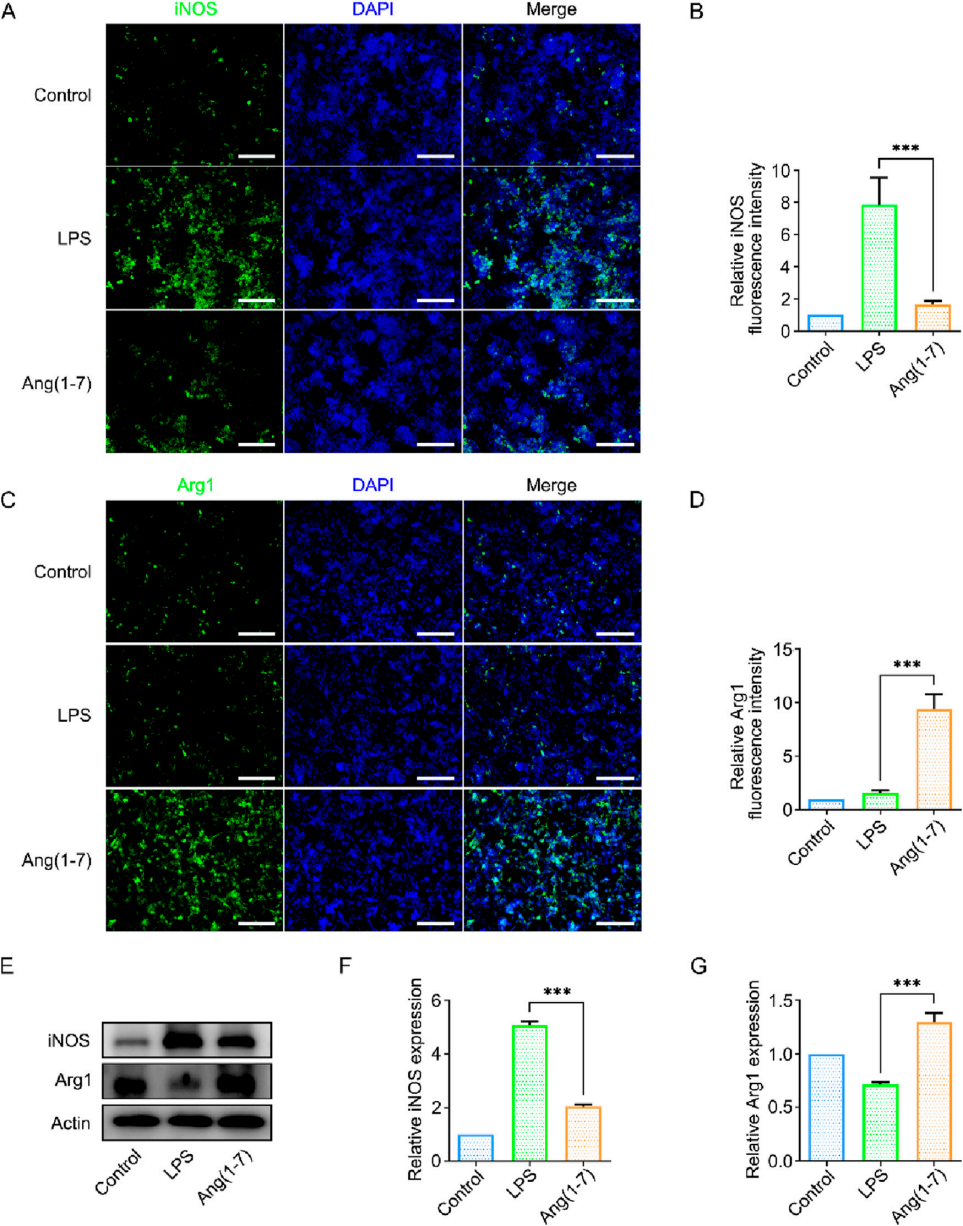
Validation of the Effect of Ang-(1–7) on BV2 Cell Polarization *In Vitro*. **(A)** Immunofluorescence images of iNOS (green) in cells from different treatment groups, scale bar = 100 μm. **(B)** Quantification of relative fluorescence intensity of iNOS. **(C)** Immunofluorescence images of Arg1 (green) in cells from different treatment groups, scale bar = 100 μm. **(D)** Quantification of relative fluorescence intensity of Arg1. **(E)** Western blot images of iNOS and Arg1 protein expression in different treatment groups. **(F)** Quantification of iNOS expression levels in different treatment groups. **(G)** Quantification of Arg1 expression levels in different treatment groups.

Immunofluorescence staining results indicated that in an inflammatory environment, the presence of Ang-(1–7) led to fewer M1-polarized microglia and more M2-polarized microglia (Figures 3A–D). In addition, this study examined the expression levels of iNOS and Arg-1 proteins in BV2 cells from different treatment groups. The Western blot (WB) results showed a trend consistent with the immunofluorescence findings. Compared to the LPS group, the Ang-(1–7) group exhibited reduced expression of iNOS, a marker of M1 cells, and increased expression of Arg-1, a marker of M2 cells (Figures 3E–G). This indicates that Ang-(1–7) promotes the polarization of microglia towards the M2 phenotype, which suppresses inflammatory responses and improves the local microenvironment at the injury site.

### 3.6 NT-3 promotes neural stem cell differentiation into neurons in vitro

**FIGURE 4:**
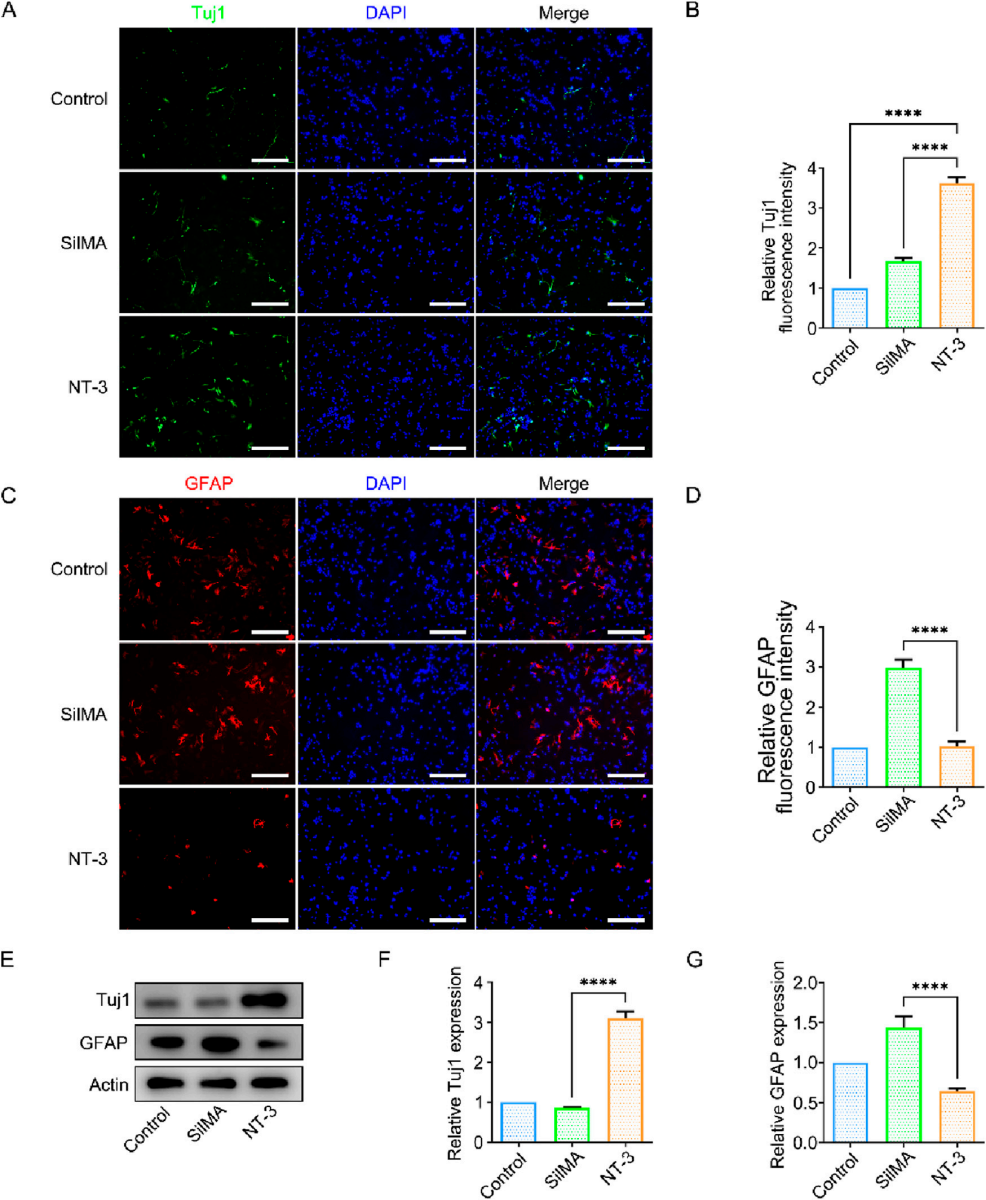
Effect of NT-3 on Neural Stem Cell Differentiation. **(A)** Immunofluorescence im-ages of Tuj-1 (green) in cells from different treatment groups, scale bar = 100 μm. **(B)** Quantification of relative fluorescence intensity of Tuj-1. **(C)** Immunofluorescence images of GFAP (red) in cells from different treatment groups, scale bar = 100 μm. **(D)** Quantification of relative fluorescence intensity of GFAP. **(E)** Western blot images of Tuj-1 and GFAP protein expression in different treatment groups. **(F)** Quantification of Tuj-1 expression levels in different treatment groups. **(G)** Quantification of GFAP expression levels in different treatment groups.

The differentiation direction of neural stem cells is a critical factor in spinal cord in-jury repair. The survival and differentiation of neural stem cells determine the extent of neural repair. Neural stem cells have the potential to differentiate into three lineages: astrocytes, neurons, and oligodendrocytes. Among these, astrocytes and neurons are the most important cell types affecting spinal cord injury repair. I In most cases, exogenous neural stem cells predominantly differentiate into astrocytes rather than neurons, both *in vivo* and *in vitro*, which hinders neural function repair. In this study, neural stem cells were co-cultured with NT-3-loaded SilMA hydrogel using Transwell inserts. Neural stem cells cultured without hydrogel were used as the control group. Immunofluorescence staining was performed using the neuron-specific marker β-III tubulin (Tuj-1) and astrocyte-specific marker glial fibrillary acidic protein (GFAP). The results showed a higher proportion of Tuj-1-positive cells and a lower proportion of GFAP-positive cells in the experimental group compared to the control group (Figures 4A–D). This indicates that the NT-3-loaded SilMA hydrogel promotes a higher differentiation rate of neural stem cells into neurons while reducing their differentiation into astrocytes. This suggests that NT-3 plays a significant role in directing neural stem cell differentiation towards neurons, which is beneficial for spinal cord injury repair. Furthermore, Western blot analysis was performed to verify the expression of Tuj-1 and GFAP proteins in the cells. The results showed that the expression of Tuj-1 in the NT-3-loaded SilMA hydrogel group was significantly higher than in the control group and the SilMA hydrogel group, while the expression of GFAP was lower (Figures 4E–G). This confirms that NT-3 at the experimental concentration can regulate the differentiation of neural stem cells towards neurons and reduce their differentiation into astrocytes. These findings validate the results obtained from the immunofluorescence experiments.

### 3.7 Enhanced microglia polarization and neural regeneration with DPSH in vivo

**FIGURE 5:**
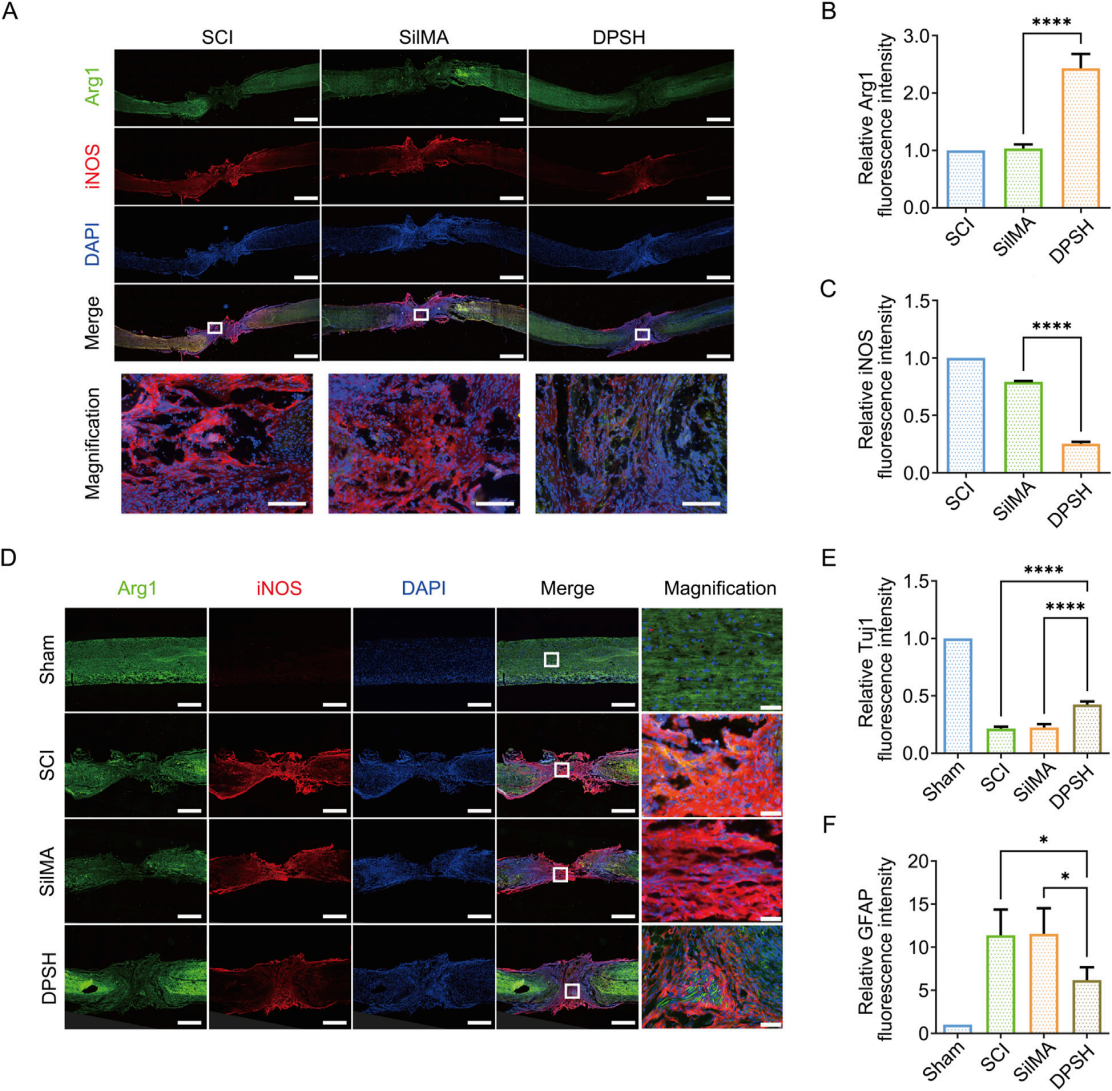
Immunofluorescence Staining and Quantitative Analysis of Spinal Cord in Mice 8 Weeks Post-SCI. **(A)** Immunofluorescence staining of Arg-1 (green) and iNOS (red) in the spinal cord of mice from different treatment groups 8 weeks post-SCI, scale bar = 500 μm. **(B)** Quantification of Arg-1 fluorescence intensity in the spinal cord of mice from different treatment groups. **(C)** Quantification of iNOS fluorescence intensity in the spinal cord of mice from different treatment groups. **(D)** Immunofluorescence staining of Tuj-1 (green) and GFAP (red) in the spinal cord of mice from different treatment groups 8 weeks post-SCI, scale bar = 500 μm. **(E)** Quantification of Tuj-1 fluorescence intensity in the spinal cord of mice from different treatment groups. **(F)** Quantification of GFAP fluorescence intensity in the spinal cord of mice from different treatment groups.

The local inflammatory response after spinal cord injury is a critical factor influencing the repair process. *In vivo* experiments have shown that SilMA hydrogel loaded with Ang-(1–7) promotes the differentiation of microglia into the M2 phenotype, thereby reducing the inflammatory response and creating a favorable microenvironment for spinal cord injury repair. I Immunofluorescence staining of spinal cord tissue cryosections revealed that the proportion of Arg-1-positive cells in the group treated with DPSH loaded with Ang-(1–7) and NT-3 was significantly higher than in the injury group and the SilMA hydrogel group, while the proportion of iNOS-positive cells was significantly lower (Figures 5A–C). This indicates that the DPSH promotes the polarization of microglia to the anti-inflammatory M2 phenotype and reduces M1 polarization at the in-jury site *in vivo*, thereby mitigating the inflammatory response and creating a conducive microenvironment for spinal cord injury repair. These findings suggest that the DPSH not only supports the structural integrity of the spinal cord but also actively modulates the immune environment, promoting better outcomes in spinal cord injury re-pair.

Neurons and synapses are fundamental structures for neural function. The differentiation of neural stem cells into neurons and the regeneration of axons are essential for neural function repair. However, changes in the local microenvironment of spinal cord injury, as well as the differentiation of astrocytes and the formation of glial scars, impede neural regeneration. Reducing scar formation is crucial for spinal cord injury repair and functional recovery. To observe the differentiation of endogenous stem cells and the ultimate outcome of spinal cord injury repair, spinal cord tissue was collected 8 weeks post-injury for protein extraction. Immunofluorescence staining was also performed on spinal cord tissue cryosections, with Tuj-1 staining marking early neurons and GFAP staining marking astrocytes. The results indicate that in the central injury area, the pro-portion of Tuj-1-positive cells in the group treated with DPSH loaded with Ang-(1–7) and NT-3 was higher than in the SCI group and the SilMA hydrogel group, while the proportion of GFAP-positive cells was lower (Figures 5D–F). This suggests that more neurons were regenerated in the DPSH group, demonstrating that NT-3 loaded in the DPSH promotes the differentiation of neural stem cells into neurons rather than astrocytes. This reduction in astrocyte differentiation and subsequent glial scar formation is beneficial for axon regeneration and the establishment of neural networks, leading to the restoration of spinal cord function.

### 3.8 SilMA hydrogel loaded with Ang-(1–7) and NT-3 PLGA microspheres promotes Mo-tor function recovery in mice

To investigate the effects of hydrogel scaffolds on spinal cord injury repair in mice, the study included a sham surgery control group (sham), a spinal cord transection model group (SCI), a group treated with SilMA hydrogel alone (SilMA), and a group treated with DPSH loaded with Ang-(1–7) and NT-3 in PLGA microspheres (4D-SilMA), with six mice in each group. After establishing the complete spinal cord transection injury model, the mice exhibited complete loss of hindlimb motor function. Over time, varying degrees of recovery were observed across the groups. The Basso Mouse Scale (BMS) was used to assess the recovery of hindlimb motor function on days 1, 7, 14, 21, 28, 35, 42, 49, and 56 post-surgery.

**FIGURE 6:**
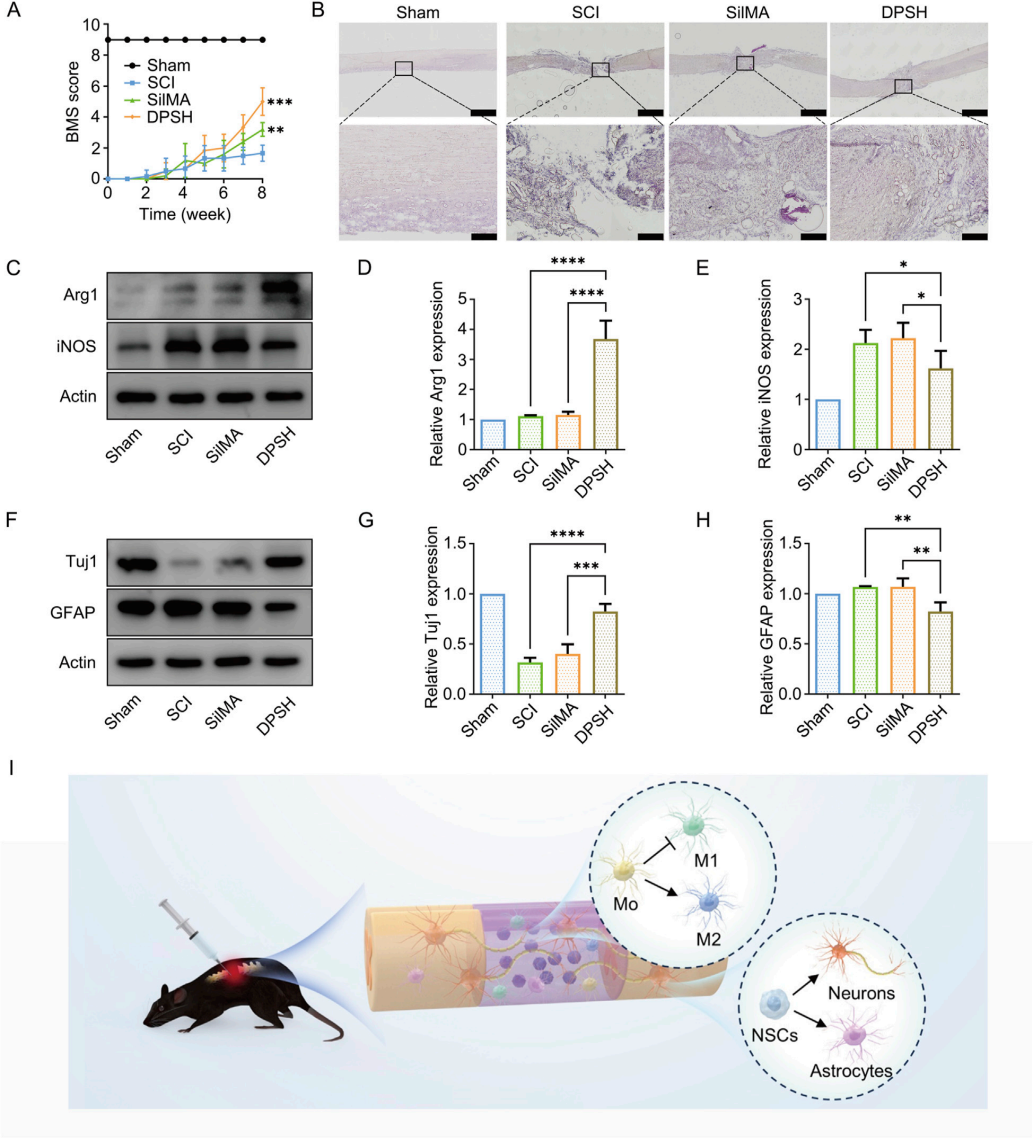
Validation of Motor Function and WB Quantitative Analysis in Mice 8 Weeks Post-SCI. **(A)** BMS scores of mice from different groups 8 weeks post-SCI. **(B)** HE staining results of spinal cord in mice from different groups 8 weeks post-SCI. **(C)** Western blot images of Arg-1 and iNOS protein expression in the spinal cord of mice from different treatment groups. **(D)** Quantification of Arg-1 expression in the spinal cord of mice from different treatment groups. **(E)** Quantification of iNOS expression in the spinal cord of mice from different treatment groups. **(F)** Western blot images of Tuj-1 and GFAP protein expression in the spinal cord of mice from different treatment groups. **(G)** Quantification of Tuj-1 expression in the spinal cord of mice from different treatment groups. **(H)** Quantification of GFAP expression in the spinal cord of mice from different treatment groups. **(I)** Schematic diagram of 4-Dimensional SilMA Hydrogel *in vivo* repair of spinal cord injury.

The results showed that on the first day after surgery, all groups of mice exhibited no autonomous hindlimb movement, with a BMS score of 0, indicating successful and stable modeling of the spinal cord transection. By the eighth week post-surgery, the BMS score of the DPSH group loaded with Ang-(1–7) and NT-3 reached 6, significantly higher than the scores of the SCI group and the SilMA hydrogel group (Figure 6A). This suggests that the DPSH scaffold significantly promotes the recovery of motor function following spinal cord injury.

### 3.9 DPSH promotes spinal cord tissue structure recovery

To investigate the recovery of spinal cord tissue in different groups following injury, hematoxylin and eosin (H&E) staining was performed on spinal cord sections from the mice. The staining results are shown in Figure 6. Sham Group: The spinal cord structure was intact without any defects, with clear boundaries between gray and white matter, abundant neurons, and complete structure. SCI Group: H&E staining revealed that the normal structure of the spinal cord was disrupted, with either incomplete or no connection at the injury site. There were numerous cavities within the spinal cord tissue and a disorganized structure, along with significant scar tissue at the injury site. SilMA Hydro-gel Group: The spinal cord continuity was partially restored with smaller scar tissue compared to the SCI group, but the structure remained disorganized, and many cavities were still present (Figure 6B). DPSH Group: The spinal cord morphology showed significant improvement compared to both the SCI group and the SilMA hydrogel group. The tissue had fewer cavities and inflammatory cells, and the cell arrangement was more orderly. These results indicate that the DPSH loaded with Ang-(1–7) and NT-3 significantly enhances the structural recovery of spinal cord tissue after injury, reducing tissue cavities and inflammation while promoting a more organized cellular structure.

### 3.10 DPSH modulates immune response and promotes neural differentiation *in vivo*

To validate the effects of DPSH on spinal cord injury repair, protein ex-traction from the injury site was performed for Western blot analysis. Results showed that the expression level of Arg-1 protein in mice treated with DPSH loaded with Ang-(1–7) and NT-3 was significantly higher than in the SCI and SilMA hydrogel groups. Conversely, the expression level of iNOS was significantly lower (Figures 6C–E). This indicates that the DPSH reduces M1 microglia and increases anti-inflammatory M2 microglia at the injury site, supporting the conclusion that it effectively modulates the immune response to promote a favorable environment for spinal cord repair.

Further validation of DPSH’s effects on neural stem cell differentiation and spinal cord injury repair was conducted through Western blot analysis. The expression level of Tuj-1 protein in mice treated with DPSH loaded with Ang-(1–7) and NT-3 was significantly higher than in the SCI and SilMA hydrogel groups, while the expression level of GFAP was significantly lower (Figures 6F–H). This trend aligns with immunofluorescence results, confirming that more cells at the injury site differentiated into neurons rather than astrocytes. These findings further support that DPSH promotes neuronal differentiation, reduces astrocyte formation, and aids in spinal cord injury repair and functional recovery.

## 4 Conclusion

The DPSH scaffold, with sustained-release Ang-(1–7) and NT-3-loaded PLGA microspheres, meets the requirements of a tissue engineering material for SCI treatment. It matches the mechanical properties and microstructure of the spinal cord and exhibits good biocompatibility. The study results indicate that the DPSH can release Ang-(1–7) to promote microglial M2 polarization, reduce inflammation, aid cell survival and regeneration, and sustain NT-3 release to promote NSC neuronal differentiation, reduce astrocyte differentiation, decrease glial scar formation, and reconstruct neural connections. This facilitates spinal cord function recovery and ultimately restores motor function in SCI mice.

## Data Availability

The original contributions presented in the study are included in the article/supplementary material, further inquiries can be directed to the corresponding authors.
